# Cognitive Behavioral Therapy Lowers Elevated Functional Connectivity in Depressed Adolescents

**DOI:** 10.1016/j.ebiom.2017.02.010

**Published:** 2017-02-16

**Authors:** Shayanti Chattopadhyay, Roger Tait, Tiago Simas, Adrienne van Nieuwenhuizen, Cindy C. Hagan, Rosemary J. Holt, Julia Graham, Barbara J. Sahakian, Paul O. Wilkinson, Ian M. Goodyer, John Suckling

**Affiliations:** a Department of Psychiatry, University of Cambridge, Cambridge, UK; b Cambridge and Peterborough Foundation NHS Trust, Cambridge, UK; c MRC/Wellcome Trust Behavioral and Clinical Neurosciences Institute, University of Cambridge, Cambridge, UK; d Department of Psychology, Columbia University, New York, NY, USA; e Department of Psychiatry, University of Oxford, Oxford, UK

**Keywords:** Depression, Adolescence, Resting-state, Functional connectivity, Fronto-limbic, Cortical thickness

## Abstract

Imaging studies have implicated altered functional connectivity in adults with major depressive disorder (MDD). Whether similar dysfunction is present in adolescent patients is unclear. The degree of resting-state functional connectivity (rsFC) may reflect abnormalities within emotional (‘hot’) and cognitive control (‘cold’) neural systems. Here, we investigate rsFC of these systems in adolescent patients and changes following cognitive behavioral therapy (CBT). Functional Magnetic Resonance Imaging (fMRI) was acquired from adolescent patients before CBT, and 24-weeks later following completed therapy. Similar data were obtained from control participants. *Cross-sectional Cohort*: From 82 patients and 34 controls at baseline, rsFC of the amygdala, anterior cingulate cortex (ACC), and pre-frontal cortex (PFC) was calculated for comparison. *Longitudinal Cohort*: From 17 patients and 30 controls with longitudinal data, treatment effects were tested on rsFC. Patients demonstrated significantly greater rsFC to left amygdala, bilateral supragenual ACC, but not with PFC. Treatment effects were observed in right insula connected to left supragenual ACC, with baseline case-control differences reduced. rsFC changes were significantly correlated with changes in depression severity. Depressed adolescents exhibited heightened connectivity in regions of ‘hot’ emotional processing, known to be associated with depression, where treatment exposure exerted positive effects, without concomitant differences in areas of ‘cold’ cognition.

## Highlights

• Adjunct study to a large randomized multicentre UK trial of conversational therapies
• Robust pre-processing methods reduced head motion artefacts, improving upon those undertaken by the majority of studies.
• Aberrant processing in ‘hot’ limbic regions without reductions in cognitive control in ‘cold’ prefrontal regions was found.
• Elevated limbic functional connectivity in patients was lowered towards values seen in controls following CBT.

This study demonstrates that unlike depressed adults, adolescents have aberrant brain connectivity in regions associated with emotional processing without concomitant differences in areas associated with cognitive control. Moreover, cognitive behavioral therapy restored such aberrant brain connectivity. The brain is thus malleable to conversational therapy, with the degree of change related to improvement in symptoms. This gives the hope that kindling effects – whereby episodes of illness increase vulnerability to further episodes – can be arrested by treatment before the occurrence of indelible changes in brain structure.

## 1 Introduction

Major depressive disorder (MDD) is a leading cause of disability (Whiteford et al., 2015). Amongst adolescents, point prevalence is 6–9% (Reivich et al., 2013), with a 25% lifetime prevalence by the end of adolescence (Kessler et al., 2001). Depressed adolescents demonstrate concurrent functional impairments in cognitive and social areas, and high rates of personality disorders, suicide, self-harm, and substance abuse (Harrington, 2001). Around 33% of diagnosed adult cases originate in adolescence (Goodyer et al., 2011). MDD can arise during adolescence from psychosocial stress factors, differences in epigenetic and genetic susceptibilities, and glucocorticoid vulnerabilities that lead separately or in combination to imbalances between bottom-up emotional (‘hot’) processing and top-down cognitive (‘cold’) processing, triggering lasting alterations in brain maturation trajectories (Hagan et al., 2015).

Blood oxygenation level dependent (BOLD) sensitive functional magnetic resonance imaging (fMRI) acquired during stimuli-free acquisition is the basis for constructing resting state functional connectivity (rsFC) networks of synchronous, spontaneous brain activity. Evidence from fMRI in depressed adults provides strong support for disruption of the fronto-limbic system, a key component of the ‘hot’ emotional processing system (Anand et al., 2009). However, the ACC has also been implicated in ‘cold’ cognition (Shackman et al., 2011). Specifically, MDD is associated with dysfunction of the anterior cingulate cortex (ACC)-pallidostriatal-thalamic-amygdala circuit, which forms part of the cortico-limbic mood-regulating circuit (Anand et al., 2009), with increased subgenual ACC (sgACC)-default mode network (DMN) and thalamus-DMN rsFC in patients (Greicius et al., 2007). Adolescent patients also demonstrate increased sgACC-insula, and sgACC-amygdala rsFC (Connolly et al., 2013).

A corresponding decrease in areas involved in ‘cold’ cognitive control, such as the PFC, is seen in adult patients (Anand et al., 2005). Reduced rsFC has also been detected in the ACC, insula, amygdala, and frontal pole (Veer et al., 2010). Whether these connectivity findings are present in adolescents with MDD is less clear, although adolescent patients have exhibited decreased rsFC between sgACC and supragenual ACC, insula, and parts of the frontal and temporal cortices (Cullen et al., 2009). Given that diagnostic characteristics are the same across all ages, we hypothesise that these findings will be replicated in this young age range prior to treatment.

National Institute for Health and Care Excellence (NICE) guidelines in the United Kingdom for the treatment of depression in adolescence recommend psychological therapies such as cognitive behavioral therapy (CBT) as first-line treatment, with or without the antidepressant fluoxetine (NICE, 2015). There is evidence from imaging that either treatment is associated with restoring fronto-limbic connectivity in adults (Gudayol-Ferré et al., 2015).

This study is an analysis of rsFC in adolescent patients with MDD enrolled in the Improving Mood with Psychoanalytic and Cognitive Therapies (IMPACT) clinical trial (Goodyer et al., 2011). As MDD is associated with pathological increases in physiological activity in regions of ‘hot’ emotional processing, and reduced in activity in regions of ‘cold’ cognitive control, we hypothesized that, prior to treatment with CBT, adolescent MDD patients would show hyper-connectivity in limbic structures and hypo-connectivity in regions connected to the pre-frontal cortex. We further hypothesized that CBT would be associated with normalising rsFC patterns in patients towards those seen in controls. Ameliorating aberrant connectivity associated with depressive illness early in the course of the disorder may pre-empt more atypical developmental changes to brain structure and related functions, thereby reducing the risk of recurrence and relapse.

## 2 Materials and Methods

### 2.1 Participants

The IMPACT trial was a pragmatic, single-blind, randomized controlled trial with the primary hypothesis that specialised psychological treatments had more enduring clinical effects in maintaining reduced depression symptoms compared with specialist clinical care (Goodyer et al., 2011). Enrolled patients from 16 Child and Adolescent Mental Health Services (CAMHS) clinics in the UK satisfied DSM-IV criteria for unipolar MDD.

The MR-IMPACT study recruited IMPACT participants from East Anglia and North London, and conducted an MRI assessment prior to randomisation (Hagan et al., 2013). Those randomized to CBT were invited to return for a second MRI assessment following completion of their treatment around 24 weeks later (Range of follow-up time for patients: 17.14–51 weeks, standard deviation: 9.27). To be eligible for the post-treatment assessment, participants should have attended at least 6 out of 20 scheduled appointments: median number attended was 8, with a mean interval of 16 days between sessions. Control participants were recruited from local schools and screened for the absence of current depressive illness by requiring a score ≤ 5 on the self-report Short Moods and Feelings Questionnaire (SMFQ) (Sharp et al., 2006). Two MRI assessments were scheduled 24 weeks apart.

The State-Trait Anxiety Inventory (STAI) scale was used to assess current anxiety state using STAI-S and long-term anxiety trait with STAI-T at both MRI assessments (Spielberger et al., 1970), with higher scores indicating greater anxiety levels. Exclusion criteria for all participants included: alcohol or drug dependence, generalized learning problems, pregnancy or breastfeeding, concurrent medication use that could adversely interact with SSRIs (patients only), and MRI contraindications (Hagan et al., 2013). Participants and their families gave signed informed consent. Ethical approval was provided by the Cambridgeshire 2 Research Ethics Committee (Reference: 09-H0308-168), following the Declaration of Helsinki.

In total, 168 participants 11–17 years old were enrolled into MR-IMPACT: 128 patients (34 males, 94 females) and 40 controls (11 males, 29 females). From these, 108 patients undertook only the pre-treatment (baseline) MRI assessment, of which 26 were excluded, along with 6 controls. Reasons for exclusion are shown in Fig. S1. Thus, 82 patients (18 males, 64 females) and 34 healthy controls (7 males, 27 females) were used for case-control comparisons: this was the *cross-sectional sample* (Table 1).

The 20 remaining patients had both baseline and post-treatment (follow-up) MRI assessments, with 3 excluded (Fig. S1). 33 controls had scans separated by a similar interval, of which 3 were excluded (Fig. S1), leaving 17 patients and 30 controls in the *longitudinal sample* (Table 2). The independence of the patients in the cross-sectional and longitudinal samples is an important feature of the analysis strategy; the *cross-sectional sample* findings were used as a mask for the *longitudinal sample* analyses.

### 2.2 MRI Acquisition and Processing

MRI scanning took place on a Siemens 3T Trim Trio scanner at the Wolfson Brain Imaging Centre, University of Cambridge, UK. BOLD-sensitive echo-planar images (EPI) were acquired at baseline and follow-up assessments, whilst participants lay awake with eyes closed. EPI scans were 8 min 56 s long with 256 whole-brain images collected. Experimental details were previously published (Hagan et al., 2013).

EPI images were processed to correct for head motion during acquisition using the speedypp algorithm from the BrainWavelet Toolbox (BWT) (www.brainwavelet.org), according to the established protocol (Patel et al., 2014). All data were non-linearly transformed to the standard stereotactic space of the MNI152 template using the Advanced Normalisation Tools (ANTs) (Avants et al., 2009). Once processed, images were overlaid with the Automated Anatomical Labelling (AAL) atlas defining 116 regions of interest (ROIs) (Tzourio-Mazoyer et al., 2002).

Mean DVARS, the average root-mean-square variance across all brain voxels of volume-to-volume difference in percent BOLD signal change, and translations and rotations about orthogonal axes were used to test for between-group differences in head motion using *t*-tests (assuming unequal variances). Eight participants were excluded as their variation of motion exceeded the range − 2 > mean DVARS > 2 (Patel et al., 2014).

### 2.3 Functional Connectivity in ‘Hot’ and ‘Cold’ Systems

**Fig. 1:**
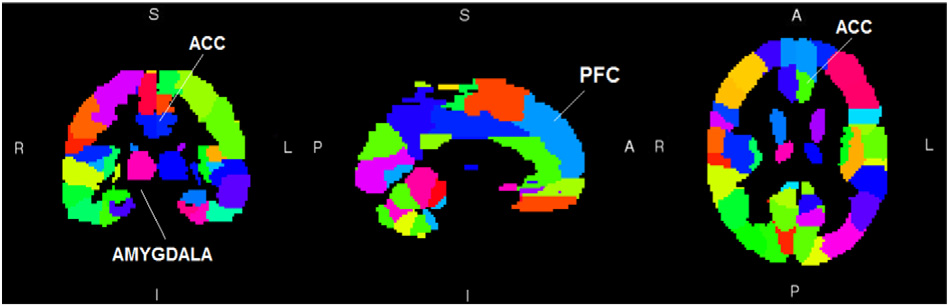
Seed regions investigated in the study. The amygdala, ACC, and PFC regions of the AAL atlas were chosen as seed regions based on previous literature to investigate case-control differences in rsFC.

Based on previously reported fronto-limbic alterations in depression, amygdala and ACC regions of the AAL atlas were chosen as seed regions to investigate case-control differences in rsFC with the *cross-sectional sample*, as they are those associated with the ‘hot’ emotional system. Bilateral PFC regions corresponding to Brodmann area 9 were chosen as seed regions indicative of the ‘cold’ cognitive system (Fig. 1), based on previous studies (Mayberg et al., 1999). MDD has been associated with specific parts of the ACC (Connolly et al., 2013, Cullen et al., 2009), therefore the ACC was sub-divided into subgenual and supragenual regions, superior and inferior to the line connecting the frontal pole to the genu of the corpus callosum, respectively.

For each seed region separately, using FMRIB's Software Library's (FSL's) FMRI Expert Analysis Tool (FEAT) tool (www.fmrib.ox.ac.uk), a univariate general linear model (GLM) was regressed at each intracerebral voxel, with the average time-series of the seed region as the independent variable and the voxel time-series as the dependent variable, to estimate rsFC with the corresponding *Z*-statistic. Between-group differences in rsFC of the *cross-sectional sample* were tested by GLM across the entire brain parenchyma. For seed regions associated with significant between-group effects, the effect of CBT on the rsFC in the *longitudinal sample* was assessed across the whole-brain by a group (patients and controls) by time (baseline and 24-week scan) interaction, which was interpreted as the treatment effect, using a two-way mixed effects GLM.

Age and gender were included as covariates in all models. All image-based statistical inference was undertaken with the FEAT or FMRIB's Local Analysis of Mixed Effects (FLAME) software on spatially extended statistics with a cluster-forming voxel threshold of z > 2.3 followed by a family-wise error rate (FWER)-corrected cluster significance threshold of *p* < 0.05.(Worsley, 2001) Significant interactions were examined graphically to determine their direction.

### 2.4 Relationship of Connectivity to Symptoms

Within brain areas demonstrating significant between-group effects in the *cross-sectional sample*, the relationship of rsFC to SMFQ, STAI-S, and STAI-T was tested in separate GLMs. The relationship between the change in mean rsFC and change in symptom scores (SMFQ) relative to baseline SMFQ was tested in areas demonstrating treatment effects in the *longitudinal sample*.

Statistical analyses were conducted in R (version 3.3.0). The threshold for significance was *p* < 0.05.

### 2.5 Cross-sectional Sample: Demographics

Patient (N = 82) and control (N = 34) participants did not differ significantly in age (t(50.32) = − 0.16, *p* = 0.88), gender (χ_1_^2^ = 0.03, *p* = 0.87), handedness (t(68.51) = − 1.44, *p* = 0.15), or IQ (t(32.28) = − 1.09, *p* = 0.28). As expected, SMFQ, STAI-S, and STAI-T scores were significantly higher for the depressed participants (*p* < 0.0001). Full details are given in Table 1, and medication status of patients in Table S1A.

### 2.6 Cross-sectional Sample: Between-group Differences in rsFC

Significant between-group differences in mean DVARS and rotation about the z-axis were found (Table S2A), although effective correction for these effects was applied during preprocessing (Patel et al., 2014).

No significant between-group differences were observed in rsFC to the right amygdala, bilateral sgACC, and bilateral PFC seed regions.

**Fig. 2:**
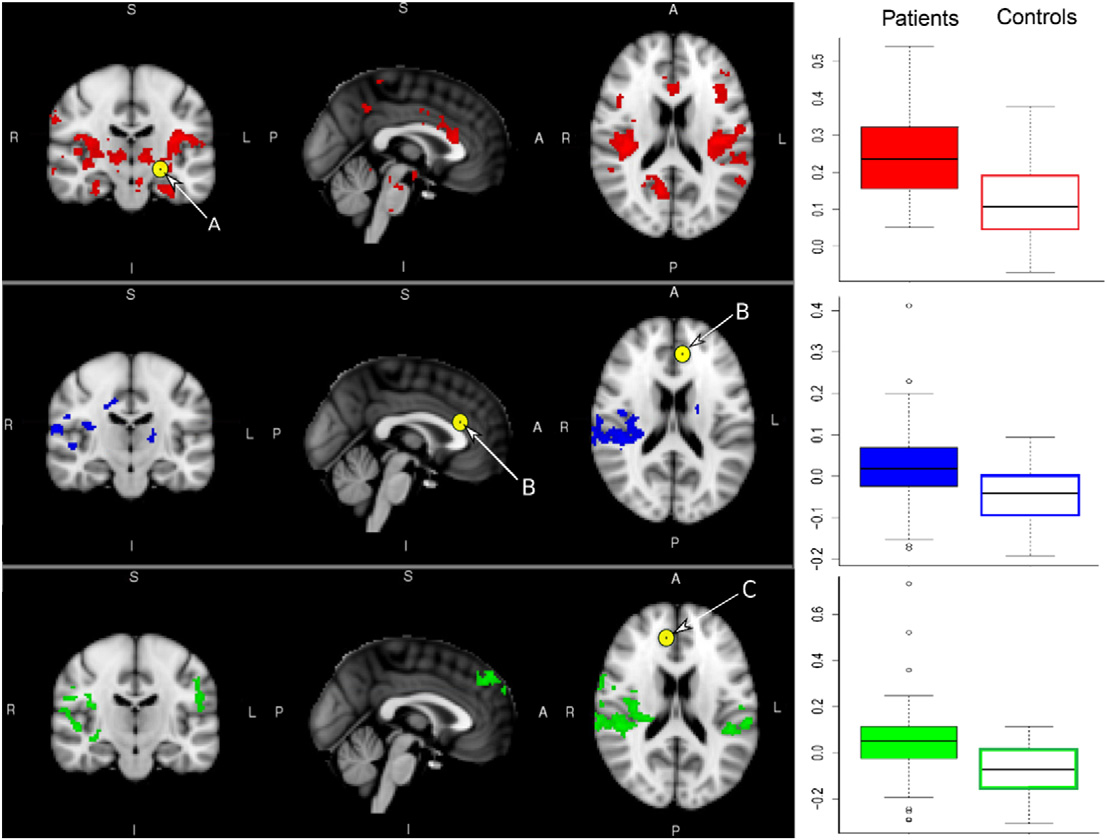
Significant case-control differences (MDD > Controls) in rsFC to the left amygdala, left supragenual ACC and right supragenual ACC seed regions respectively were found for the *cross-sectional sample* before patients with MDD underwent cognitive behavioral therapy (CBT) sessions (N = 116, *p* < 0.05). A: Left Amygdala seed; B: Left Supragenual ACC seed, C: Right Supragenual ACC seed.

Patients exhibited greater rsFC between the left amygdala seed region and the insula, supragenual ACC, thalamus, hippocampus, right amygdala, right parietal areas, right angular and lingual gyri, right putamen, precuneus, and right PCC (Fig. 2, Table S3). Patients also exhibited greater rsFC between the left supragenual ACC seed region and the right planum temporale, right parietal operculum, right post-central gyrus, right PCC, left thalamus, and bilateral insula (Fig. 2, Table S3), as well as between the right supragenual ACC seed region in the right pre-central gyrus, supramarginal gyrus, frontal pole, insula, bilateral post-central gyrus, and superior temporal gyrus (Fig. 2, Table S3).

### 2.7 Cross-sectional Sample: Relationships to Symptoms

For depressed adolescents, there were no significant relationships between rsFC in regions demonstrating a between-group difference to any seed region and symptoms measured with SMFQ, STAI-S, or STAI-T.

### 2.8 Longitudinal Sample: Demographics

Patients (N = 17) in the *longitudinal sample* were not included in the *cross-sectional sample*, although controls (N = 30) were drawn from the same individuals. The longitudinal patient samples did not differ on age (t(35.83) = − 0.40, *p* = 0.70), gender (χ_1_^2^ = 0.04, *p* = 0.84), handedness (t(41.11) = − 0.06, *p* = 0.95), or IQ (t(4.83) = − 0.45, *p* = 0.67) compared to controls (Table 2). As anticipated, SMFQ, STAI-S, and STAI-T scores were significantly higher for patients (Table 2). Medication status is given in Table S1B. There were no significant differences in demographics and symptoms between patients in the cross-sectional and longitudinal samples (t(25.99) = − 0·04, *p* = 0.97).

### 2.9 Longitudinal Sample: Treatment Effects

**Fig. 3:**
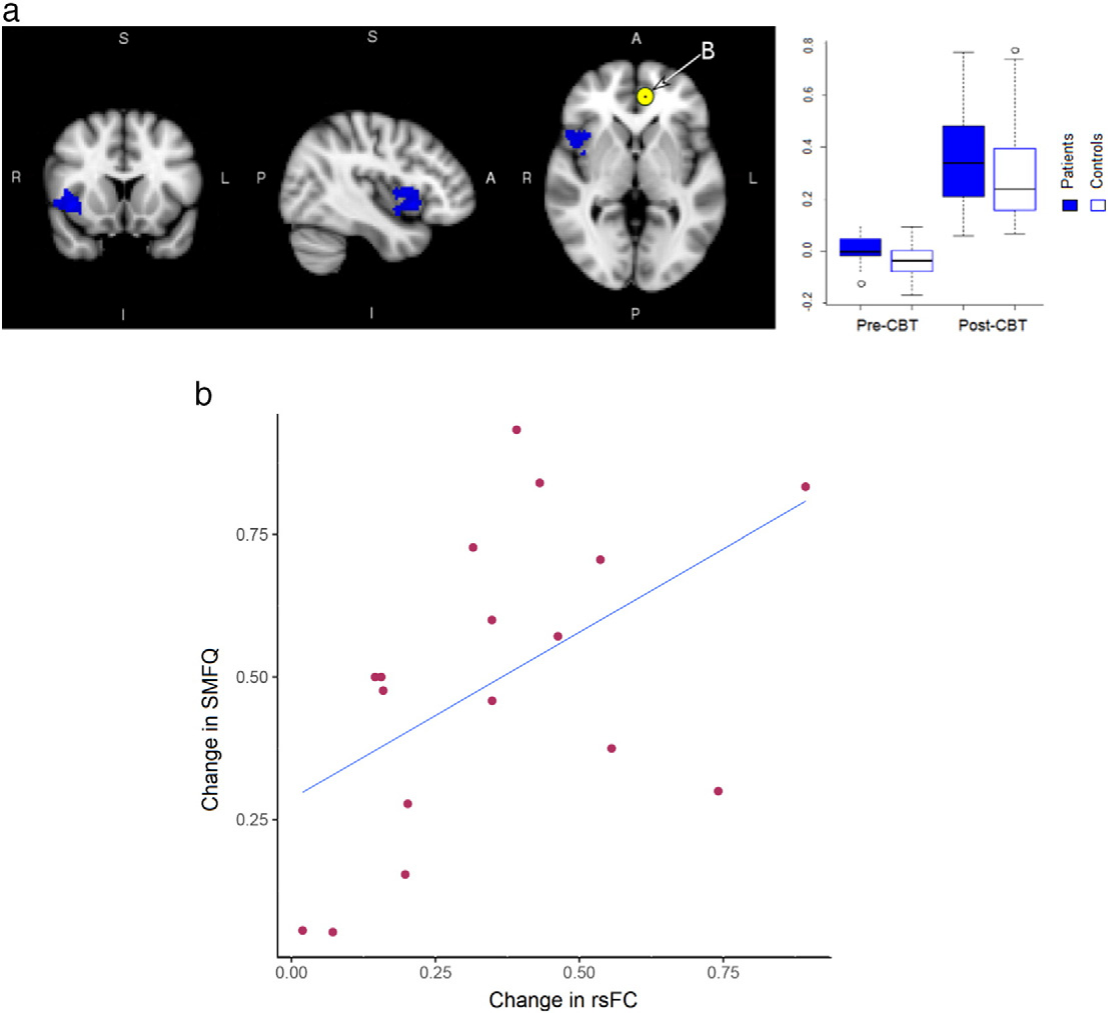
a. Treatment effects seen with left supragenual ACC seed in right insula in the *longitudinal sample*. b. Significant relationship between change in SMFQ vs change in rsFC (relative to baseline SMFQ) to left supragenual ACC seed was noted in the patient group (F(1, 13) = 5.44, *p* = 0.04).

Connectivity estimates associated with seed regions where between-group effects were seen (i.e., left and right supragenual ACC, left amygdala), were tested for treatment effects across the entire parenchyma in the *longitudinal sample*. There were no significant treatment effects in regions connected to left amygdala or right supragenual ACC. However, significant effects of treatment were seen with rsFC to the left supragenual ACC seed in right insula (F(1, 43) = 5.28; *p* = 0.02) (Fig. 3). rsFC in both patients and controls was increased in the post-CBT scan. Post-hoc tests of between-group effects in the *longitudinal sample* indicates that the significant between-group effect at baseline (F(1, 43) = 6.92; *p* = 0.01) was significantly different from the between-group difference following CBT (F(1, 43) = 1.05; *p* = 0.31).

### 2.10 Longitudinal sample: Relationships to Symptom Change

Patients in the *longitudinal sample* demonstrated a significant reduction in symptom severity after receiving CBT: baseline, mean SMFQ = 16.71 ± 4.90; post-treatment, mean SMFQ = 8.88 ± 5.12 (F(1, 30) = 21.18; *p* = 7.14 × 10^− 5^). In regions showing significant treatment effects with the left supragenual ACC seed, there was also a significant relationship between changes in rsFC and changes in SMFQ post-treatment, relative to baseline SMFQ values (F(1, 13) = 5.44, *p* = 0.04, effect size = 0.52) (Fig. 3B), as well as STAI-T (F(1, 13) = 5.26, *p* = 0.04, effect size = 0.51), but non-significant when related to STAI-S (F(1, 13) = 0.11, *p* = 0.77, effect size = 0.08).

## 3 Discussion

This study investigates the effect of treatment on rsFC in neural regions indexing the ‘hot’ emotional, and ‘cold’ cognitive systems in adolescents with MDD. As hypothesized, depressed adolescents had increased rsFC of parts of the ‘hot’ limbic system relative to controls. However, no difference was found with connectivity to the ‘cold’ PFC. These results suggest a specific pathology to the limbic system following the onset of symptoms. Whether this represents an illness-related effect or a putative marker prior to illness emergence cannot be determined from these cross-sectional findings. Evidence of such abnormalities has been found in high-risk individuals. Hyper-connectivity in left temporal cortex, insula, ACC, medial orbitofrontal PFC, and ventromedial PFC was observed in 6-month-old infants born to mothers with prenatal maternal depressive symptoms, similar to patterns observed in depressed adolescents and adults (Qiu et al., 2015). Development of ‘hot’ emotional processing and ‘cold’ cognitive processing systems may occur at different rates during adolescence, and asynchronous trajectories may have adverse consequences for mental health and contribute to the emergence of MDD (Hagan et al., 2015). In this study, elevated rsFC of the ‘hot’ (limbic) system in the absence of differences in the ‘cold’ (pre-frontal) system implies that depressive symptoms may emerge from physiologically overactive bottom-up cortical regions with a loss of influence from their top-down counterparts.

Our study is aligned with previous research on adults with depression showing altered rsFC in areas which may be critical for mood regulation (Anand et al., 2005). The right insula exhibited significantly greater connectivity with left amygdala and bilateral supragenual ACC seed regions. Altered insular rsFC has been shown in depressed adolescents and adults (Connolly et al., 2013, Veer et al., 2010. The right anterior insula may play a role in adaptively switching from rumination to interoceptive awareness (Horn et al., 2010). Hyper-connectivity between the ACC and insula has proved to be more equivocal in adolescents; both elevated and reduced rsFC to the subgenual ACC and the insula have been reported (Connolly et al., 2013, Cullen et al., 2009). Such differences may reflect variation in experimental details, or in sample characteristics, such as medication use. Here, 30 of 82 patients were medicated. However, comparing only those on medication to controls (Fig. S5) or medication-naïve to controls (Fig. S4) led to similar patterns of increased rsFC to left amygdala and bilateral supragenual ACC compared to healthy controls. Length of time on medication may also be a factor, but was not available.

Early life stress has been associated with markedly elevated rates of MDD in child, adolescent, and adult cohorts, and may therefore affect brain function, leading to observable differences in rsFC prior to onset of a first episode (Henje Blom et al., 2015).

All patients had improved symptoms following CBT. Successful treatment was associated with normalisation of rsFC (Fig. 3). The sensitivity of this change in right insula was corroborated by a normalisation of rsFC along with a reduction in symptoms scores; larger alterations in rsFC led to reductions in symptoms (Fig. 3B). Changes in insula activity have been shown to take place following various treatments, including medication, deep brain stimulation, vagus nerve stimulation, and mindfulness training, indicating a role for this region in mediating treatment response (McGrath et al., 2013). This suggests that cognitive mechanisms involved in recovery involve the inhibition of pathological rumination and the re-instigation of interoceptive processing and monitoring of bottom-up physiological signals. Significant time effects were observed for all seed regions which did not overlap with treatment effects. These may reflect maturation changes with time or accommodation of participants to the MRI environment, or both.

The significant baseline group differences in rsFC and treatment effects observed are in adjacent, but distinct areas of the right insula. This may be due to a reduction in power in the longitudinal analysis, or that regions with small baseline differences, undetectable in a whole-brain analysis after appropriate multiple comparisons correction, are more amenable to CBT; that is, only areas of the limbic system that are not extensively damaged are recoverable.

These findings must be interpreted with limitations in mind. The study was a parallel group, longitudinal design typical for assessing treatment effects. However, the control group were healthy adolescents rather than patients randomized to placebo, due to ethical concerns of withholding treatment and the difficulty of providing a placebo conversational therapy. Therefore, we cannot conclude whether improvements were due to CBT or non-specific effects of improvement in symptoms. Patients were recruited from the IMPACT effectiveness trial with potential participants approached sequentially in clinical settings. The sample thus reflects the local patient population, subject to biases in consenting. Male participants constituted around 25% (25/91), which differs from the 2:1 female:male ratio more widely observed in adolescents with depression (Murray, 2013). Furthermore, the follow-up patient sample was limited in size.

The 11–17 years age range covers the period during which brain maturation is significant. As treatment effects were the focus of the study, effects of age and gender, although modelled, were not investigated here. There were no significant demographic differences between groups. Significant between-group differences in some head motion parameters were found. Hence, motion cannot be completely ruled out as a source of between-group rsFC differences. However, in a comparison of a subset of participants with no significant between-group difference in DVARS, patterns of increased rsFC in the limbic system were sustained (Fig. S3).

In summary, this study investigated rsFC differences in adolescents with MDD compared to healthy controls. Depressed adolescents demonstrated greater rsFC in limbic regions associated with ‘hot’ emotional processing in the absence of differences in regions associated with ‘cold’ cognition; that is, an imbalance between these systems. This is possibly due to the relative speeds of maturation of the two systems (Hagan et al., 2015). Coupled with similar results from elsewhere, rsFC is a potential marker for MDD in adolescence. Neurobiologically, CBT normalized aberrant rsFC patterns in the limbic system of adolescents with MDD. Symptom recovery may at least in part be associated with a ‘cooling’ of ‘hot’ emotional brain systems, and their restoration is a key component of the mechanism of action of therapeutic interventions, such as CBT.

## Funding Sources

UK Medical Research Council (MRC) (grant: G0802226), National Institute for Health Research (grant: 06-05-01), the Department of Health, Behavioral and Clinical Neuroscience Institute (University of Cambridge), the latter being jointly funded by the MRC and the Wellcome Trust. Additional support received from the Cambridge Biomedical Research Centre. SC is supported by the University of Cambridge Overseas Trust and CONACyT: Data collection and analyses.

## Conflicts of Interests

SC, RT, TS, AV, CCH, and RJH report no biomedical financial interests or potential conflicts of interests. JG reports grants from MRC during the conduct of the study. BJS reports personal fees from Cambridge Cognition, Peak (Brainbow), Mundipharma, Lundbeck, Otsuka, and grants from J&J, outside the submitted work. POW reports personal fees from Lundbeck and Takeda, and grants from MRC and CLAHRC-EoE, outside the submitted work, and is an interpersonal psychotherapy supervisor and trainer. IMG reports grants from NIHR-HTA, grants from Wellcome Trust Strategic Award, outside the submitted work. JS reports grants from MRC, National Institute for Health Research, Wellcome Trust or MRC, during the conduct of the study; grants from GlaxoSmithKline plc, personal fees from GlaxoSmithKline plc, outside the submitted work.

## Author Contributions

SC, JS, and AV drafted the manuscript, with further edits provided by RT, TS, CCH, RJH, JG, BJS, POW, and IMG. SC conducted the literature search and designed all figures. SC, RT, and TS conducted the analyses. AV, CCH, RJH, and JG were involved in data collection. POW, IMG, and JS designed the study.

## Figures and Tables

**Table 1:** Demographic and clinical characteristics of depressed and healthy adolescents from the *cross-sectional sample* (N = 116).

Characteristic	MDD adolescents n = 82 Mean (SD)	Control adolescents n = 34 Mean (SD)	*p*-value (*p* < 0.05 for significance)
Age (years)	15.69 (1.12)	15.73 (1.44)	0.875
Range	13.48–17.96	12.14–17.73	
Gender (Male/Female)	18/64	7/27	0.871
Handedness (Edinburgh Handedness Inventory (46))	54.51 (58.84)	70.56 (52.67)	0.154
IQ (from 17 patients, 34 controls)	97.35 (10·73)	100.82 (10.79)	0.285
SMFQ score	18.02 (4.89)	2.65 (1.97)	*p* < 0.0001
STAI State score	45.95 (10.00)	28.82 (6.76)	*p* < 0.0001
STAI Trait score	59.78 (8.02)	30.59 (6.58)	*p* < 0.0001

**Table 2:** Demographic and clinical characteristics of the depressed and healthy adolescent participants from the *longitudinal sample* (N = 47).

Characteristic	MDD adolescents n = 17 Mean (SD)	Control adolescents n = 30 Mean (SD)	*p*-Value (*p* < 0.05 for significance)
Age at first scan (years)	15.42 (1.37)	15.59 (1.47)	0.690
Range	12.89–17.56	12.14–17.73	
Age at second scan (years)	16.07 (1.34)	16.24 (1.46)	0.695
Range	13.41–18.17	12.92–18.30	
Gender (Male/Female)	3/14	6/24	0.844
Handedness (Edinburgh Handedness Inventory (46))	66.47 (41.82)	67.30 (55.26)	0.954
IQ (from 5 patients, 30 controls)	98.60 (14.40)	101.67 (11.15)	0.669
SMFQ score at first scan	16.71 (4.90)	2.57 (1.81)	7.46 × 10^− 10
SMFQ score at second scan	8.88 (5.12)	2.37 (1.85)	7.62 × 10^− 5
STAI State score at first scan	48.24 (11.33)	28.90 (6.91)	1.63 × 10^− 6
STAI State score at second scan	35.59 (10.13)	26.10 (10.48)	0.002
STAI Trait score at first scan	61.94 (9.08)	30.50 (6.91)	1.44 × 10^− 12
STAI Trait score at second scan	44.12 (10.48)	28.37 (6.49)	1.00 × 10^− 5

## Appendix A Supplementary data

Supplementary material Image 1
