# Supplementary material for: Cognitive Behavioral Therapy Lowers Elevated Functional Connectivity in Depressed Adolescents
Source: eBioMedicine. 2017 Feb 16;17:216–22. doi: 10.1016/j.ebiom.2017.02.010 (PMC5360581; doi:10.1016/j.ebiom.2017.02.010)

**SUPPLEMENTARY MATERIAL**

The cohort in this study is derived from the Magnetic Resonance Imaging adjunct study to the Improving Mood with Psychoanalytic and Cognitive Therapies clinical trial (MR-IMPACT; 128 MDD, 40 controls). Participants were taken from the larger study after matching for age and gender as well as correcting for head motion.

The datasets were pre-processed using the BrainWavelet Toolbox (www.brainwavelet.org). Any subjects that failed the pre-processing, had signal dropouts, excess head motion, or missing data, were excluded. In addition, MDD patients who had a co-morbid diagnosis of psychosis were excluded from our study. The various sample sizes and reasons for exclusion are shown in Figure S1.

The final sample used in the cross-sectional analysis comprised 116 participants (82 MDD; 34 controls). Demographics of this cohort are given in Table 1. No significant between-group differences were noted with respect to age (*p* = 0·87) or gender (*p* = 0·87).

Patients who completed a second scan after CBT formed the longitudinal sample with 47 participants (17 MDD; 30 controls). The demographics of this cohort are given in Table 2. No significant between-group differences were noted with respect to age at first scan (*p* = 0·69), age at second scan (*p* = 0·70), or gender (*p* = 0·84).

30 MDD patients were treated with antidepressant medications, mainly selective serotonin re-uptake inhibitors (SSRIs). The medication status of the patients is displayed in Table S1A. There were no significant differences in age (t(53·18) = 0·67, *p* = 0·51), gender (χ_1_^2^ = 3·58, *p* = 0·06), or symptom severity (t(66·01) = 0·35, *p* = 0·73) between the 52 unmedicated and 30 medicated sub-groups of the MDD participants in the cross-sectional cohort. Figures S4, S5, and S6 show differences in rsFC between medication-naïve patients and healthy controls, medicated patients vs healthy controls, and medicated patients vs medication-naïve patients respectively.

**Table S1A. Age, gender, and psychiatric medications taken by MDD patients in the cross-sectional sample.**

| **Age (years)** | **Gender** | **Medication** | **Daily dose (mg)** |
| --- | --- | --- | --- |
| 15.73 | F | Fluoxetine | 20 |
| 13.78 | F | Fluoxetine | 40 |
| 15.34 | F | Fluoxetine | 20 |
| 15.28 | F | Fluoxetine | 20 |
| 16.9 | M | Fluoxetine | 20 |
| 16.58 | F | Fluoxetine | 20 |
| 14.95 | M | Fluoxetine | 20 |
| 13.88 | M | Fluoxetine | 20-40 (Alternating) |
| 15.73 | F | Fluoxetine | 20 |
| 16.97 | F | Fluoxetine | 20 |
| 14.65 | F | Fluoxetine | 20 |
| 14.90 | F | Sertraline | 50 |
| 16.33 | F | Fluoxetine | 20 |
| 17.36 | F | Fluoxetine | 20 |
| 16.23 | F | Citalopram | 10 |
| 17.44 | M | Fluoxetine | 40 |
| 15.96 | F | Fluoxetine | 20 |
| 16.18 | F | Fluoxetine | 30 |
| 14.88 | M | Fluoxetine | 20 |
| 16.64 | F | Lamotrigine | 25 |
| 13.48 | M | Citalopram | 20 |
| 15.42 | M | Fluoxetine | 10 |
| 16.93 | F | Fluoxetine | 20 |
| 13.88 | F | Fluoxetine | 5 |
| 17.52 | F | Fluoxetine | 40 |
| 14.85 | M | Fluoxetine | 10 |
| 17.80 | F | Fluoxetine, Risperidone | 20, 0.5 |
| 17.83 | M | Fluoxetine | 20 |
| 15.43 | M | Fluoxetine | 20 |

**Table S1B.** **Age, gender, and psychiatric medications taken by MDD patients in the longitudinal sample.**

| **Age at Scan 2 (years)** | **Gender** | **Medication** | **Daily dose (mg)** |
| --- | --- | --- | --- |
| 16.96 | M | Citalopram | 40 |
| 16.34 | F | Fluoxetine | 20 |
| 14.45 | F | Fluoxetine, Risperidone | 20, 2 |
| 15.46 | F | Fluoxetine | 30 |
| 17.27 | F | Fluoxetine | 10 |
| 14.15 | F | Fluoxetine | 20 |

**Head Motion**

Head motion of all participants was measured using mean DVARS, which is the average root mean square variance across all brain voxels of frame-to-frame difference in percent signal change. DVARS between groups was compared using *t*-tests (unequal variances assumed) of mean DVARS, and translations and rotations about orthogonal axes. Significant between-group differences in mean DVARS were observed for the cross-sectional participants (t(88·04) = 2·58, *p* = 0·01). Between-group differences in displacements and rotations were non-significant for all parameters, besides rotation about z-axis (*p* = 0·01). Details of the test results are below.

**Mean DVARS**

**Table S2A. Cross-sectional sample*:* MDD vs Controls: t(88**·**04) = 2**·**58, *p* = 0**·**01**

| **Comparison** | **Direction** | **t** | **df** | **p-value** |
| --- | --- | --- | --- | --- |
| MDD vs controls | x-displacement | 0·84 | 95·61 | 0·40 |
|  | y-displacement | -0·04 | 77·35 | 0·97 |
|  | z-displacement | 0·81 | 87·27 | 0·42 |
|  | Rotation about x | 0·95 | 97·90 | 0·34 |
|  | Rotation about y | 1·77 | 82·35 | 0·08 |
|  | Rotation about z | -2·61 | 88·86 | 0·01* |

Significantly increased motion (as seen by difference in mean DVARS) in the MDD patients was observed compared to controls. The BWT pre-processing should have significantly alleviated the effects of movement, but motion cannot be completely excluded as a source of between-group difference.

**Longitudinal sample**

No significant differences in mean DVARS were observed for MDD and control participants for the pre-treatment scans (t(22.84) = 1.11, *p* = 0.28), or the post-treatment scans (t(30.15) = 0.42, *p* = 0.68). Between-group differences in displacements and rotations were non-significant for all parameters.

**Table S2B. Pre-treatment scan: MDD vs Controls: t(22**·**84) = 1·11, *p* = 0·28**

| **Comparison** | **Direction** | **t** | **df** | **p-value** |
| --- | --- | --- | --- | --- |
| MDD vs controls | x-displacement | 0·62 | 23·54 | 0·54 |
|  | y-displacement | 0·67 | 38·54 | 0·50 |
|  | z-displacement | 0·71 | 23·65 | 0·48 |
|  | Rotation about x | 0·30 | 31·73 | 0·76 |
|  | Rotation about y | 1·07 | 24·96 | 0·29 |
|  | Rotation about z | -1·29 | 28·14 | 0·21 |

**Table S2C. Post-treatment scan: MDD vs Controls: t(30**·**15) = 0·42, *p* = 0·68**

| **Comparison** | **Direction** | **t** | **df** | **p-value** |
| --- | --- | --- | --- | --- |
| MDD vs controls | x-displacement | -0·26 | 24·31 | 0·80 |
|  | y-displacement | -0·25 | 20·32 | 0·80 |
|  | z-displacement | -0·08 | 24·92 | 0·93 |
|  | Rotation about x | 0·04 | 36·10 | 0·97 |
|  | Rotation about y | -0·93 | 26·77 | 0·36 |
|  | Rotation about z | 0·15 | 25·79 | 0·88 |

**Table S3. Characteristics of significant cluster regions from the cross-sectional sample analysis.**

| **FC Differences** | **# of voxels** | ***p*-value** | **Peak region z-value** | **Peak MNI Co-ordinates (mm)** | **Key brain regions**  **(Peak region(s) in bold)** |
| --- | --- | --- | --- | --- | --- |
| ***Seed: Left Amygdala***  MDD>Control | | | | | |
| Cluster 4 | 9140 | 4·97e-25* | 4·63 | (42, 14, -10) | Right hemisphere**: insula**, parietal operculum, amygdala, putamen, brain stem;  bilateral: thalamus, hippocampus, inferior frontal gyrus, central operculum |
| Cluster 3 | 869 | 0·0003* | 3·55 | (4, 16, 28) | Right hemisphere: **ACC**;  left: ACC, juxtapositional lobule cortex |
| Cluster 2 | 535 | 0·01* | 3·12 | (10, -66, 50) | Right hemisphere: **precuneus**, lateral occipital cortex, PCC, postcentral gyrus, superior parietal lobule;  left: postcentral gyrus, precuneus, insula |
| Cluster 1 | 513 | 0·01* | 3·58 | (10, -64, 20) | Right hemisphere**: precuneus**, PCC, supracalcarine cortex, lingual gyrus, angular gyrus |
| Age Effect (Reduced rsFC with age) | | | | | |
| Cluster 1 | 61799 | 0* | 5·47 | (-36, 8, -14) | Left hemisphere: **left insula,** occipital fusiform gyrus, precentral gyrus; right hemisphere: frontal pole, inferior frontal gyrus;  bilateral: putamen, pallidum, insula, central operculum, planum temporale, lateral occipital cortex |
| ***Seed: Right Amygdala***  Age Effect (Reduced rsFC with age) | | | | | |
| Cluster 3 | 17031 | 6·64e-40* | 5·24 | (6, -56, -12) | Left hemisphere**: lingual gyrus**, insula, putamen;  bilateral: precentral gyrus, postcentral gyrus, inferior frontal gyrus, thalamus, central operculum |
| Cluster 2 | 1552 | 4·17e-07* | 3·76 | (36, 16, 56) | Right hemisphere**: middle frontal gyrus,** ACC, paracingulate gyrus |
| Cluster 1 | 429 | 0·02* | 3·49 | (-32, -66, 48) | **Left lateral occipital cortex,** angular gyrus |
| ***Seed: Left Supragenual ACC***  MDD>Control | | | | | |
| Cluster 2 | 1191 | 1·85e-06* | 3·88 | (38, -32, 16) | Right hemisphere: **planum temporale, parietal operculum, post-central gyrus, right insula,** central operculum, superior temporal gyrus, PCC, putamen |
| Cluster 1 | 474 | 0·007* | 3·58 | (-28, -2, 4) | Left hemisphere: **putamen, insula,** central operculum, thalamus |
| Age Effect (Increased rsFC with age) | | | | | |
| Cluster 1 | 780 | 0·0002* | 4·14 | (54, -34, 28) | Right hemisphere: **parietal opercular cortex, supramarginal gyrus,** planum temporale, supramarginal gyrus |
| ***Seed: Right Supragenual ACC***  MDD>Control | | | | | |
| Cluster 3 | 1909 | 2·13e-09* | 8·67 | (58, 4, 34) | Right hemisphere: **pre-central gyrus**, supramarginal gyrus, insula, superior temporal gyrus, postcentral gyrus |
| Cluster 2 | 832 | 7·41e-05* | 4·13 | (-56, -28, 12) | Left hemisphere: **planum temporale**, parietal operculum, superior temporal gyrus, middle temporal gyrus, postcentral gyrus, precentral gyrus |
| Cluster 1 | 340 | 0·04* | 1·38 | (16, 40, 52) | Right hemisphere: **frontal pole,** superior frontal gyrus, paracingulate gyrus, left frontal pole, left superior frontal gyrus |
| Age Effect (Reduced rsFC with age) | | | | | |
| Cluster 2 | 399 | 0·02* | 3·41 | (18, 46, 20) | Right hemisphere: **frontal pole,** ACC, superior frontal gyrus |
| Cluster 1 | 381 | 0·02* | 3·1 | (4, -80, -4) | Right hemisphere: **lingual gyrus,** intra-calcarine cortex; left: intra-calcarine cortex, supracalcarine cortex, occipital pole |
| Gender Effect (Females>Males) | | | | | |
| Cluster 1 | 389 | 0·02* | 4·16 | (-28, -76, -8) | Left hemisphere: **occipital fusiform gyrus**, lateral occipital cortex, lingual gyrus, inferior temporal gyrus |
| ***Seed: Left Pre-frontal Cortical Region***  Age Effect (Reduced rsFC with age) | | | | | |
| Cluster 6 | 2274 | 4·5e-09* | 3·74 | (-64, -34, 42) | Left hemisphere: **supramarginal gyrus,** post-central gyrus, precentral gyrus, middle frontal gyrus |
| Cluster 5 | 1344 | 4·83e-06* | 3·61 | (48, -30, 36) | Right hemisphere: **supramarginal gyrus**, superior parietal lobe, postcentral gyrus |
| Cluster 4 | 1050 | 6·04e-05* | 3·54 | (-6, 4, 30) | Left hemisphere: **ACC**, juxtapositional lobule cortex; right hemisphere: juxtapositional lobule cortex, middle frontal gyrus |
| Cluster 3 | 724 | 0·001* | 3·97 | (4, -52, -26) |  |
| Cluster 2 | 523 | 0·01* | 3·83 | (48, 2, 20) | Right hemisphere: **pre-central gyrus**, middle frontal gyrus, inferior frontal gyrus |
| Cluster 1 | 482 | 0·02* | 3·46 | (-40, 18, 2) | Left hemisphere: **left frontal operculum cortex**, insula, inferior frontal gyrus |
| Gender Effect (Females>Males) | | | | | |
| Cluster 1 | 456 | 0·02* | 3·65 | (8, 28, 56) | Right hemisphere: **superior frontal gyrus**, middle frontal gyrus, frontal pole, paracingulate gyrus |
| ***Seed: Right Pre-frontal Cortical Region***  Age Effect (Reduced rsFC with age) | | | | | |
| Cluster 5 | 3817 | 8·66e-13* | 4·13 | (32, 12, 8) | Right hemisphere: **insula**, precentral gyrus, juxtapositional lobule, precentral gyrus, middle frontal gyrus |
| Cluster 4 | 2921 | 1·64e-10* | 4·16 | (-64, -34, 42) | Left hemisphere: **supramarginal gyrus**, precentral gyrus, middle frontal gyrus, postcentral gyrus |
| Cluster 3 | 2224 | 1·43e-08* | 3·88 | (36, -44, 42) | Right hemisphere: **superior parietal lobule**, supramarginal gyrus, postcentral gyrus |
| Cluster 2 | 2097 | 5·96e-08* | 4·32 | (26, -74, -48) |  |
| Cluster 1 | 843 | 0·001* | 4·25 | (-26, 18, 6) | Left hemisphere: **insula**, inferior frontal gyrus, frontal operculum, frontal orbital cortex, putamen |

**Table S4. Characteristics of significant group-by-time interaction effects from the longitudinal sample analysis.**

| **FC Differences** | **# of voxels** | ***p*-value** | **Peak region z-value** | **MNI Co-ordinates**  **(mm)** | **Key brain regions**  **(Peak region(s) in bold)** |
| --- | --- | --- | --- | --- | --- |
| ***Seed: Left Supragenual ACC*** | | | | | |
| Cluster 1 | 443 | 0·02* | 4·28 | (46, 6, 4) | Right hemisphere: **central operculum**, insula, frontal operculum, inferior frontal gyrus |

**CORTICAL THICKNESS ANALYSIS**

Cortical thickness maps were constructed in native space from the T1-weighted images using the ANTs^21^ and FSL ([www.fmrib.ox.ac.uk)](http://www.fmrib.ox.ac.uk)) toolkits. Non-linear and affine warps were generated with brain extracted images mapped to the MNI152 template with the ANTs Symmetric Normalisation tool^21^.

Case-control differences in the cross-sectional cohort were tested with a GLM at all locations on the cortical surface, covarying for age and gender, at a level of significance of *p*<0·05 with a FWER correction for multiple comparisons. There were no significant differences.

**Supplementary Figures**

**Fig S1. Sample sizes and reasons for exclusion of participants.**

**
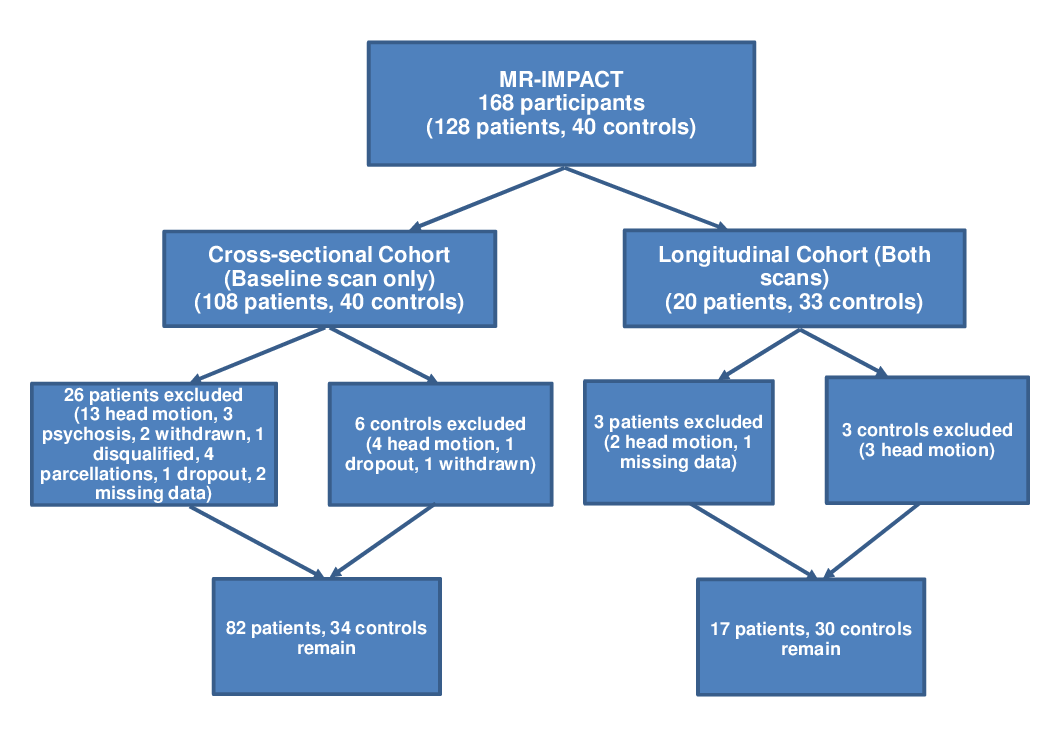
**

**Controls' rsFC Networks**

The average pattern of rsFC in healthy controls was determined by analysing the group mean effect with each seed region (Figure S2). The average rsFC to the left amygdala primarily involved the caudate, superior frontal gyrus, precuneus, PCC, and temporal lobe regions. For the right amygdala, rsFC was concentrated in the caudate, cingulate, putamen, hippocampus, and superior temporal gyrus areas. The left and right supragenual ACC showed elevated rsFC to peripheral regions and the frontal lobes. In case of the left PFC region, clusters of higher connectivity were seen in the superior frontal gyrus, caudate, thalamus, paracingulate gyrus, ACC, parahippocampal gyrus, precuneus, PCC, and inferior frontal gyrus. The right PFC region also showed high rsFC to the precuneus, PCC and superior frontal gyrus, and middle temporal gyrus.

**Supplementary Fig S2.** **Pattern of seed regions' connectivity in healthy controls (N = 34) (2**·**3 < *Z* < 10**·**8).**

| 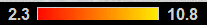  **LEFT AMYGDALA**  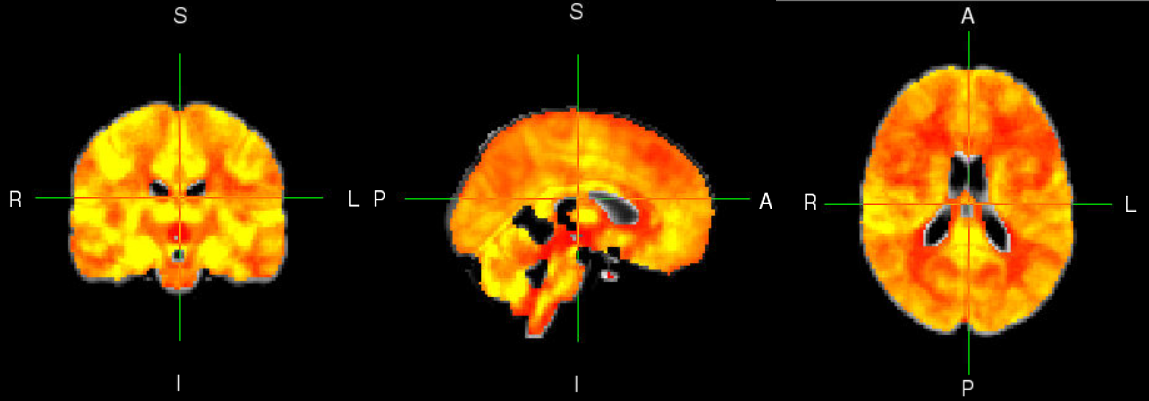 |  |
| --- | --- |
| **RIGHT AMYGDALA**  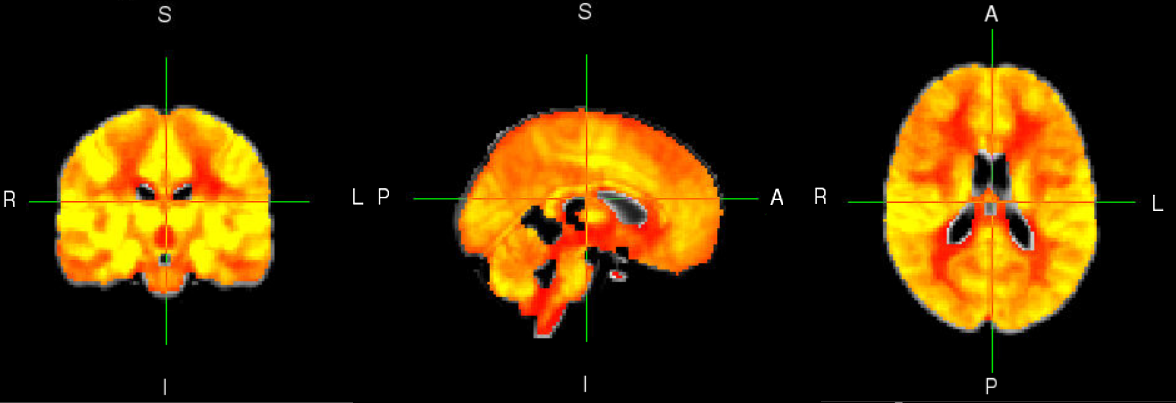  **LEFT SUPRAGENUAL ACC** |  |


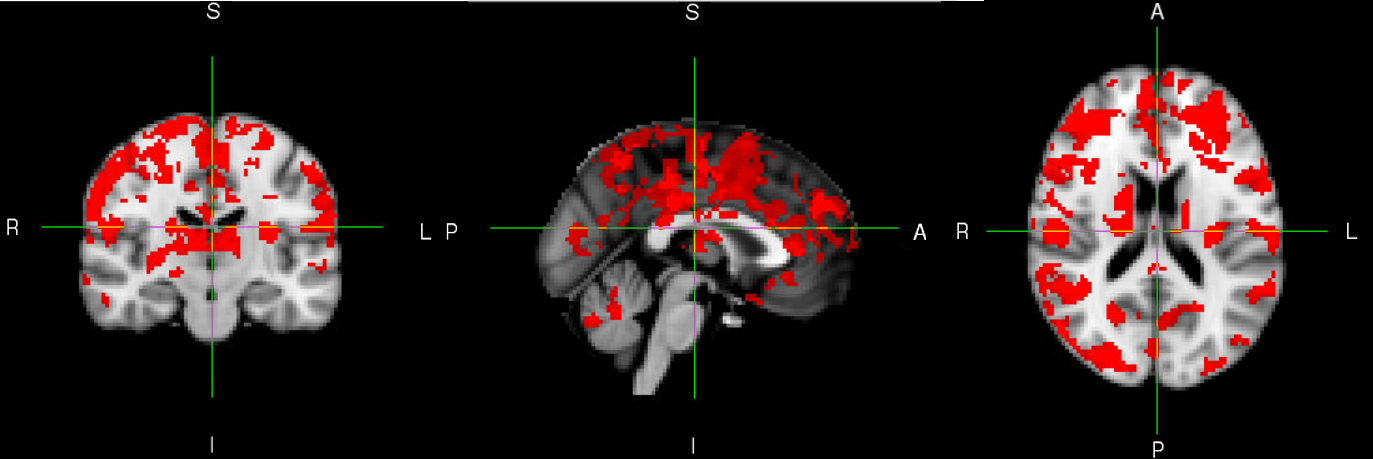


**LEFT SUBGENUAL ACC**

**
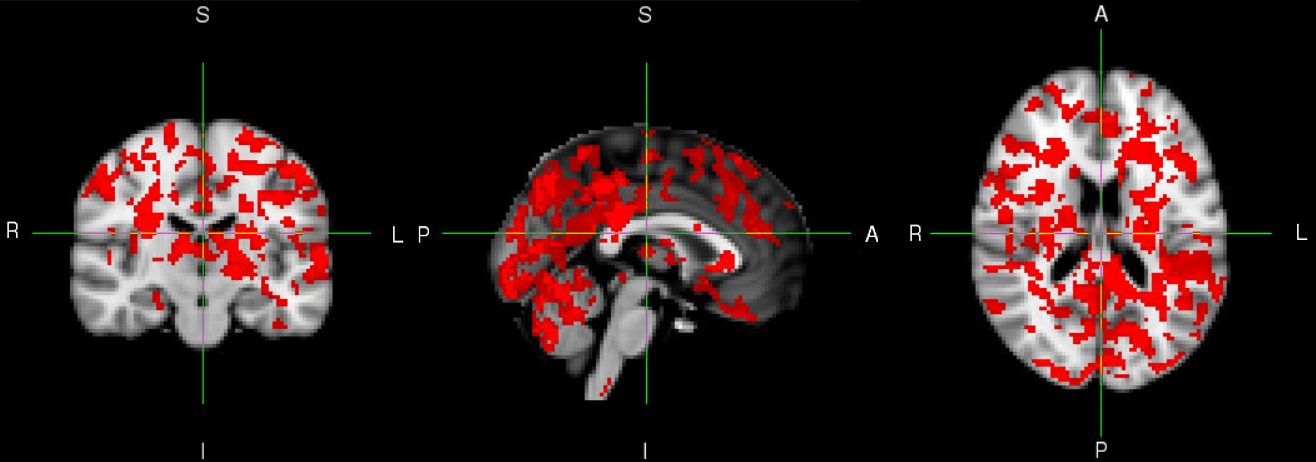
**

**RIGHT SUPRAGENUAL ACC**

**
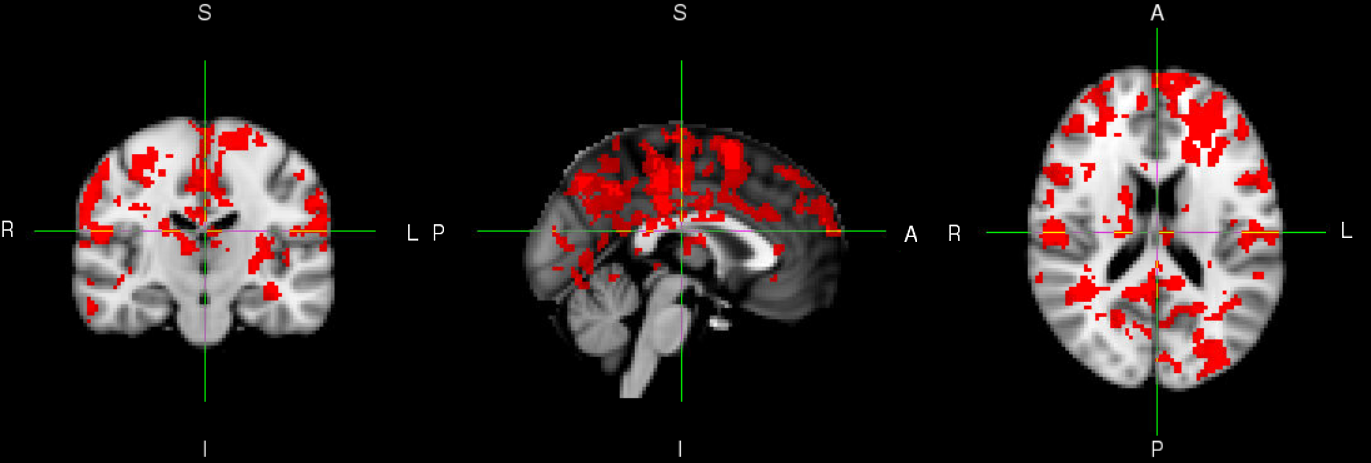
**

**RIGHT SUBGENUAL ACC**

**
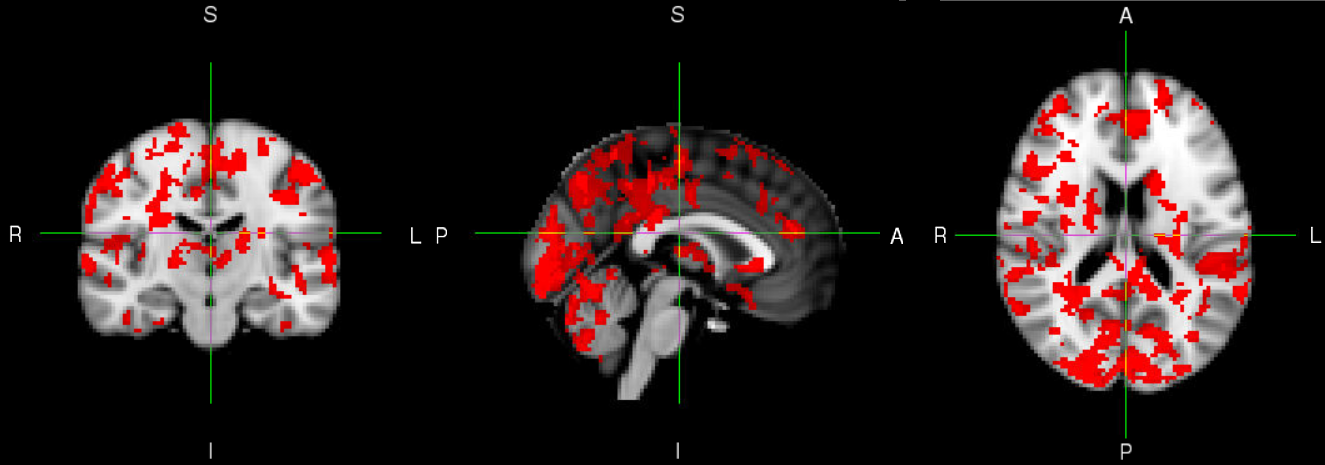
**

| **LEFT PFC REGION**  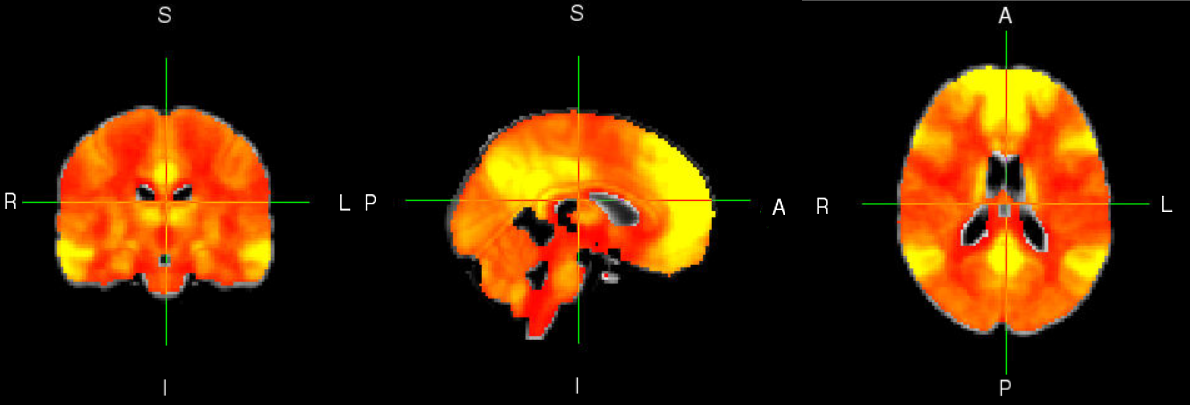  **RIGHT PFC REGION** |  |
| --- | --- |
| **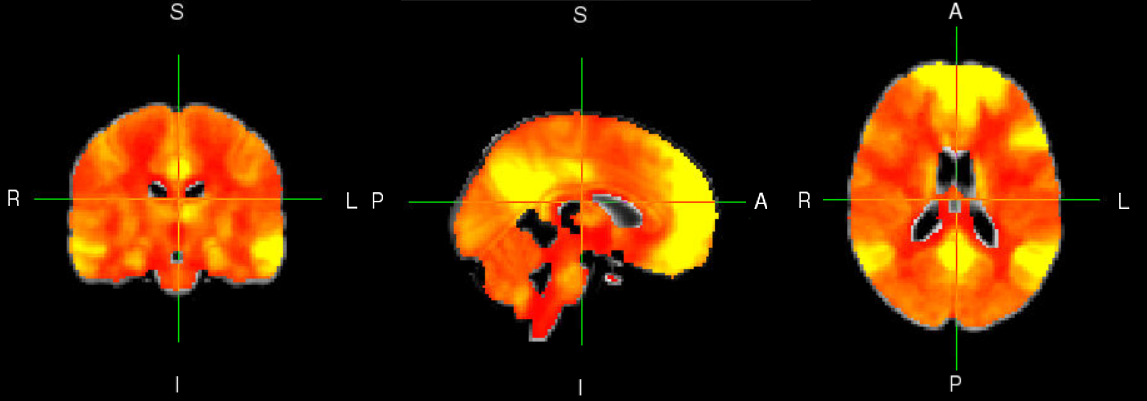** |  |

**Fig S3. Pattern of significant between-group differences in rsFC to left amygdala, left and right supragenual ACC seed regions respectively when group differences in DVARS were eliminated (*p* = 0.08). 8 patients and 1 control with DVARS > 1**·**75 standard deviations from the mean were removed to eliminate significant between-group differences in DVARS. Case-control differences corresponding to each seed region for these participants were similar to those obtained in the cross-sectional sample. Differences were absent with the PFC seed.**

***A: Left Amygdala seed; B: Left Supragenual ACC seed; C: Right Supragenual ACC seed***

**
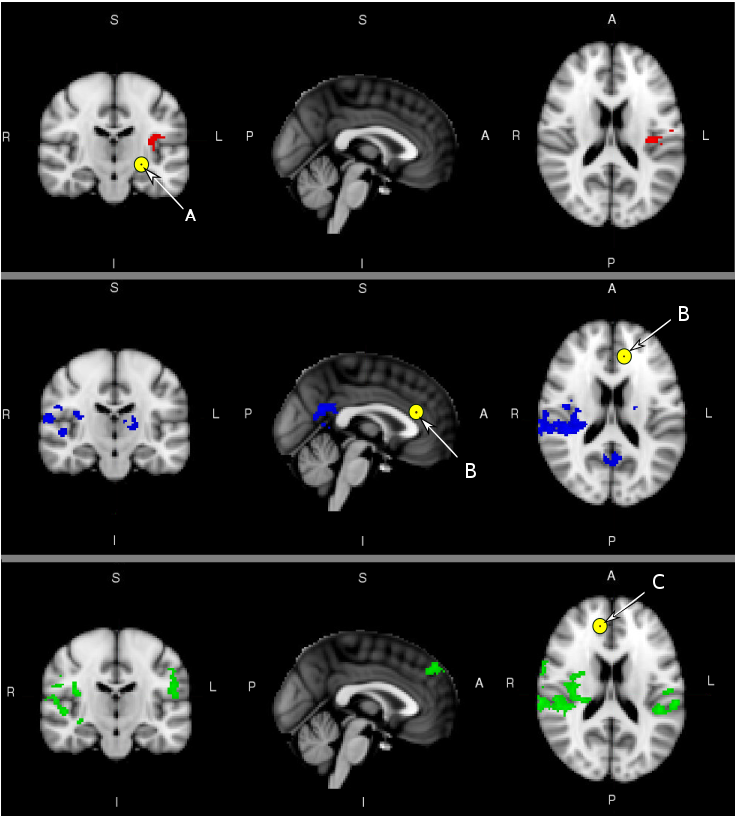
**

**Fig S4. Medication-naïve vs controls (52 vs 34) were compared using left amygdala, left and right supragenual ACC seeds respectively. The pattern of greater rsFC in patients was maintained.**

***A: Left Amygdala seed; B: Left Supragenual ACC seed; C: Right Supragenual ACC seed***


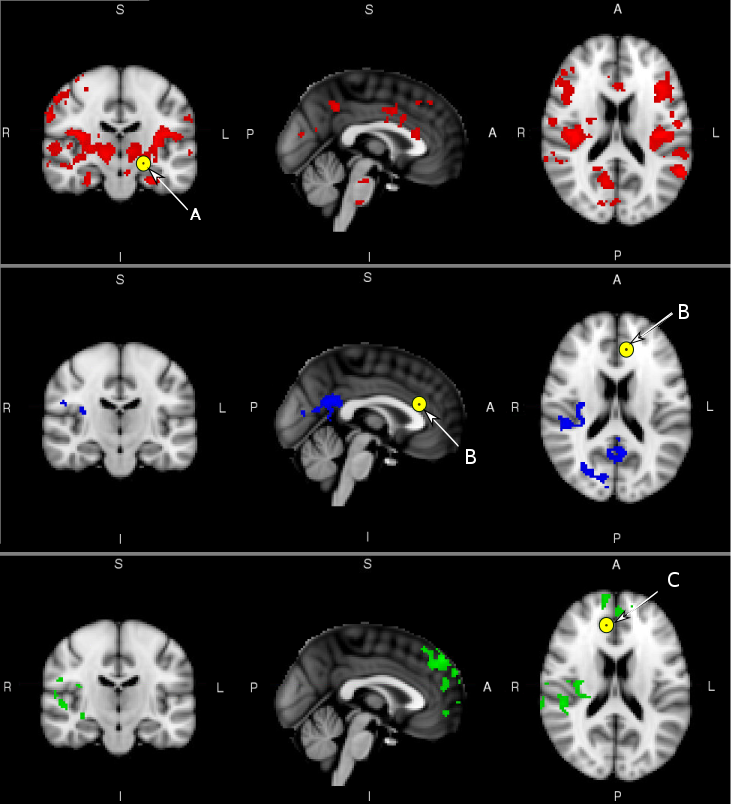


**Fig S5. Medicated MDD vs controls (30 vs 34) were compared using left amygdala, left and right supragenual ACC seeds respectively. Patients showed increased rsFC compared to controls.**

***A: Left Amygdala seed; B: Left Supragenual ACC seed; C: Right Supragenual ACC seed***

**
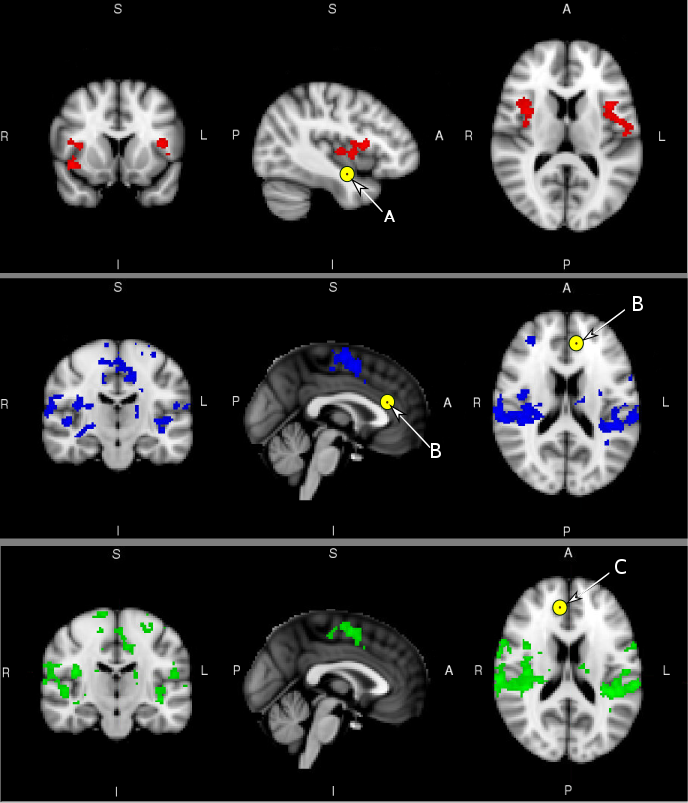
**

**Fig S6. Medicated depressed vs unmedicated MDD patients were compared using left amygdala, left and right supragenual ACC seeds respectively. Medicated MDD patients appeared to have lower rsFC compared to unmedicated patients to left amygdala and right supragenual ACC seeds.**

***A: Left Amygdala seed; C: Right Supragenual ACC seed***


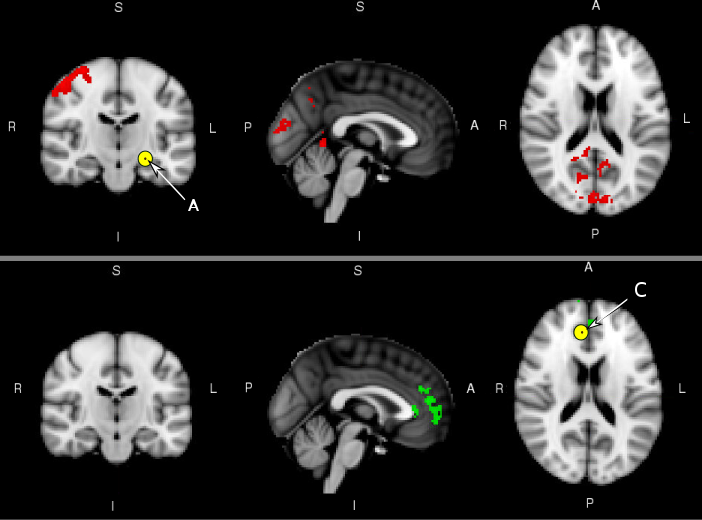


**Medicated MDD patients appeared to have greater rsFC compared to unmedicated patients for the right supragenual ACC seed in the juxtapositional lobule cortex.**


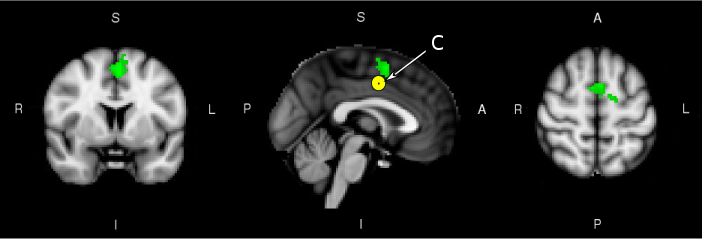

Supplement: Supplementary file 1 — Supplementary material [file mmc1.docx]
